# Scholarly Publishing, Boundary Processes, and the Problem of Fake Peer Reviews

**DOI:** 10.1177/01622439221112463

**Published:** 2022-07-17

**Authors:** Kirsten Bell, Patricia Kingori, David Mills

**Affiliations:** 1 University of Roehampton, London, England; 2 University of Oxford, Oxford, England

**Keywords:** peer review, refereed manuscripts, fake reviews, scholarly publishing, knowledge production, Retraction Watch, COPE

## Abstract

Over the past decade, the phenomenon of “fake” peer reviews has caused growing consternation among scholarly publishers. Yet despite the significant behind-the-scenes impact that anxieties about fakery have had on peer review processes within scholarly journals, the phenomenon itself has been subject to little scholarly analysis. Rather than treating fake reviews as a straightforward descriptive category, in this article, we explore how the discourse on fake reviews emerged and why, and what it tells us about its seeming antithesis, “genuine” peer review. Our primary source of data are two influential adjudicators of scholarly publishing integrity that have been critical to the emergence of the concept of the fake review: Retraction Watch and the Committee on Publication Ethics. Via an analysis of their respective blog posts, Forum cases, presentations, and best practice guidance, we build a genealogy of the fake review discourse and highlight the variety of players involved in staking out the fake. We conclude that constant work is required to maintain clear lines of separation between genuine and fake reviews and highlight how the concept has served to reassert the boundaries between science and society in a context where they have increasingly been questioned.

## Introduction

In March 2016, in her role at the time as co-editor of an interdisciplinary social science journal owned by Taylor & Francis, Kirsten Bell received an email notifying academic journal editors that the “preferred reviewer” function had been removed from their submission platforms. According to Taylor & Francis, this decision had been made as a consequence of the problem of “fake” peer reviews—authors subverting the peer review process by either recommending fake academics with email accounts they controlled or by naming real academics but providing fake email addresses for them. In the accompanying press release they circulated, Taylor & Francis explained their decision as follows:

One of the most high-profile ethical issues that has continued to be a problematic area of concern is the increase in cases of “fake reviewers.” As a responsible publisher, Taylor & Francis have been looking at ways to tackle this issue, and one of the steps we have taken to safeguard the integrity of our peer review has been to remove the “preferred reviewers” function from our ScholarOne Manuscripts and Editorial Manager sites.

Submitting authors quickly noticed the disappearance of the function and some contacted the editorial team to complain about its removal. The editors also noticed its loss: while we did not always approach recommended reviewers, we found the recommendations useful to have—although we generally searched the reviewers online to ensure they had the right content expertise and so on. It therefore seemed to us that Taylor & Francis’s actions constituted an overresponse to a minor problem—and one that seemed largely confined to journals in STEM fields. In June 2016, Bell lobbied the journal’s managing editor at Taylor & Francis about getting the reviewer function reinstated. Taylor & Francis doubled down on the claim that fake reviewers were a widespread problem, noting that their actions were in keeping with publishers, such as BioMed Central, PLoS, and Sage. It was, they reiterated, a matter of “integrity,” although they also highlighted the “harm to the reputations of editors, journals and publishers.”

In this article, we explore this phenomenon of fake peer reviews—a topic that, to date, has received little attention in the academic literature, despite its significant behind-the-scenes impacts on knowledge production within scholarly journals. The key exceptions are hyperbolic editorials published primarily in medical periodicals. One representative example titled “Fraudsters strike peer review” states: “during the past few years, the world’s biomedical journals have been struck by a small but consistent rash of falsified peer reviews, a scientific crime spree that is pushing editors and publishers to greater vigilance to secure the integrity of the research they publish” (Greene 2015, 13a). In the rare instances where the topic has been addressed by scholars of science interested in how publication ethics and academic misconduct are being conceptualized, they tend to take the phenomenon of fake reviews at face value (e.g., Jacob 2019; Biagioli and Lipmann 2020). Instead of treating fake reviews as a straightforward descriptive category, we are interested in how the fake review discourse emerged and why and what it reveals about its seeming antithesis, “genuine” peer review.

The origins of scientific peer review are typically traced to royal academies in the United Kingdom and France, although historians differ on when practices resembling contemporary peer review emerged and their early functions (see Csiszar 2018), with some arguing that their key purpose was quality control (Merton with Zuckerman 1973; Kronick 1990), and others asserting that peer review was linked to the need to locally administer licensing and censorship requirements (e.g., Biagioli 2002; Fitzpatrick 2011). Peer review only became widespread in scholarly journals and funding agencies in the period after World War II, although it has since become centrally embedded in the structure and operations of publishing and the academy more broadly (Chubin and Hackett 1990; Biagioli 2002; Shatz 2004; Fyfe et al. 2020). Described as the “alpha and omega of scientific production” (Pontille and Torny 2015, 58), peer review is today the institutionalized mechanism for distinguishing flawed, inferior and bogus studies from their sound, meritorious, innovative counterparts (Chubin and Jasanoff 1985).

Although the meanings of peer review have remained relatively stable since the postwar period, the conditions under which peer review is conducted have shifted radically in recent decades (Kaltenbrunner et al. 2021). The digital transition, the consolidation of commercial control over the scholarly publishing ecology, and the recasting of publications as “outputs,” with its attendant emphasis on journal prestige as a proxy for researcher quality, have intensified tensions between the different roles of academic publishing. Today, scholarly publications are simultaneously “a means of disseminating validated knowledge,” “a form of symbolic capital for academic career progression,” and “a profitable business enterprise” (Fyfe et al. 2017, 2). Yet, as Kaltenbrunner et al. (2021, 4) note, despite the new functions of peer review, “the political–economic organization of peer review remains understudied, both in regard to the labor it draws on and in terms of how scholarly publishing as a business mediates its epistemic workings.”

While gaps remain in our understanding of the peer review process, its imperfections have long been on display. Indeed, peer review has had critics since its inception (see Csiszar 2018). Numerous observers have highlighted a gap between the rhetoric of peer review and the empirical practices it entails (see Shapley and Roy 1984; Chubin and Jasanoff 1985; Chubin and Hackett 1990; Shatz 2004; Siler and Strang 2017). They have questioned whether “good” and “bad” science can be so readily distinguished, whether unbiased, independent peer review is possible, whether peer review supports or suppresses innovations in knowledge, and whether gatekeepers use peer review in good faith. To date, these criticisms have done little to dampen the enthusiasm for peer review, although there have been various efforts to reform it, including a growing array of experiments with open review, peer-to-peer review, post-publication review, and so on (see Fitzpatrick 2011). This is primarily because the practice remains critical to scientific “boundary-work” (Gieryn 1983): affirming the distinction between “science” and “non-science.” As Chubin and Hackett (1990) observe, peer review serves a critical symbolic and ideological function in guaranteeing the autonomy and authority of science. Yet, peer review is itself a boundary process: “a mix of communities, purposes, evidential standards, argumentative procedures, ethical precepts, theoretical frameworks, epistemic cultures, principles of fairness and the like mingle and collide in the review process” (Hackett and Chubin 2003, 8). Peer review lies at the nexus of the interests of numerous players with very different kinds of investments in it. For this reason, the concept of the fake review provides an invaluable opportunity to examine the work of peer review and the conditions under which it is carried out, serving to articulate the various social worlds in which it is enmeshed and the subjectivities embedded in the process.

Our primary source of data for the analysis that follows are two increasingly influential adjudicators of integrity in scholarly publishing: the Committee on Publication Ethics (COPE) and Retraction Watch. Formed in 1997, COPE (2000) initially consisted of a small group of UK medical journal editors and provided a “forum for meetings of editors, publishers, and others associated with the publication of biomedical journals; to encourage and promote ethical standards in medical publications.” In 2008, when COPE drafted its second code of conduct, its remit had expanded from biomedical publication ethics to publication ethics more broadly, although its principles remained essentially unchanged (Bell 2016). By 2010, it framed itself as “helping journals to get their houses in order,” and its membership had greatly expanded, primarily due to the fact that various publishers had unilaterally “signed up their entire catalogue of journal titles as COPE members” (COPE 2010). Despite its biomedical roots, COPE is the leading global arbiter of scholarly publication ethics and the primary source of “best practice” frameworks and guidelines across a range of disciplines. Its expansion speaks to the growing professionalization of publication ethics, which is now a thriving industry with its own cadre of self-trained experts, charities, and networks of stakeholders that have developed guidance, accreditation programs, and training workshops in response to growing institutional concerns about academic misconduct (Jacob 2019).

In contrast to COPE, Retraction Watch is a self-appointed publishing watchdog-cum-media outlet that stands (quite intentionally) outside the ecology of scholarly publishing itself (see Oransky 2020). Founded in 2010 by science journalists Ivan Oranksy and Adam Marcus, Retraction Watch investigates and publicizes journal retractions, seeing these as a lens into the scientific process. Its daily blog posts and active Twitter feed highlight new stories or topical additions to a database of more than 20,000 retracted papers across numerous disciplines. By 2022, Retraction Watch had approximately 70,000 Twitter followers, and it is regularly cited by other scientists and mainstream media outlets. Its blog is overseen by the Centre for Scientific Integrity, a registered US not-for-profit, and it has received philanthropic funding^1^ to employ researchers and build its retraction database. Like other new media organizations, Retraction Watch’s investigations benefit from the energy and contributions of self-appointed scientific sleuths, detectives, and whistleblowers within the academy. Its combative investigative journalism, combined with engaged moral commentary, is part of the organization’s success. As Gross and Harmon (2016) note in their study of how science has been changed by the internet, Retraction Watch is strengthened by their willingness to editorialize—especially their use of irony and sarcasm—as a tool to “transform the moral order so that it conforms more closely to the acknowledged norms of science” (p. 169). Thus, unlike traditional science journalism, they are not merely reporting the news but are instead “deeply engaged in the politics of change, a politics with important consequences for what counts as knowledge” (Gross and Harmon 2016, 157).

In March 2021, we conducted searches of the COPE website using the terms “fake reviews,” “compromised reviews,” and “12-12”^2^ and analyzed all materials produced, including Forum cases, video footage, and guidelines. In the same month, we conducted a search of the term “fake review” on Retraction Watch’s website. This produced sixty articles after the removal of those mentioning fake reviews in passing but not focusing on this phenomenon or summaries of prior articles in Retraction Watch (e.g., their “Weekend Reads”). We also conducted extensive searches of Retraction Watch’s database of retracted articles to contextualize their coverage of fake reviews on their blog. All the materials our search produced were subject to repeated open readings and cross-referenced with each other to try and build a genealogy of the fake review discourse. Our approach drew on the principles of ethnographic content analysis (Altheide 1987) and was guided by various categories of interest (e.g., geographies of authenticity, rhetorical characterizations of compromised peer review processes), alongside those that iteratively emerged during the analysis. Here, we extend recent critical examinations of fakeness and fakery to explore this as a feature of knowledge production (see Kingori and Douglas-Jones 2020; Jacob 2020).

## The Rise of the Fake Review Discourse

The problem of “compromised” peer review first appeared on the COPE website in 2011 as part of its Forum, a “self-help group for editors” (Jacob 2019, 80). Brought to COPE’s attention by a journal editor, the case (11–27) involved an author providing fake email addresses for real potential reviewers, with the editor’s suspicions being raised after a search of one of the reviewers revealed they had a different email address than the one provided by the author. The editor tested this by sending review requests to the institutional address of the reviewers listed in addition to the addresses provided by the submitting author, which led to the review request being turned down by the “real” reviewer, while the “bogus” reviewer returned their review within hours. Highlighting this as a “serious form of misconduct [that] may even be criminal,” COPE (2011) advised the editor to contact the author’s institution and inform them of the situation. This advice was repeated virtually word-for-word in case 12–12 the following year. Involving an editor who had “flagged up concerns with the preferred reviewers being suggested and their comments,” the author had listed the same preferred reviewers for numerous papers, all possessing non-institutional email addresses. The editor’s suspicions were duly raised because “comments were being returned very quickly (within 24 hours) and were often brief and positive, largely restricted to grammatical errors. All preferred reviewers favoured immediate acceptance or acceptance subject to minor revisions” (COPE 2012). After determining that the reviewers were “faked,” the editor confronted the author, who admitted to creating dummy accounts or using those of close associates. Shortly after the publication of case 12–12, COPE received a third case raising similar issues regarding “‘fake’ reviewers” (case 12–16).

Interestingly, case 12–12 is identified by COPE as its initial exposure to fake reviews. Frequently referenced on the site’s materials discussing “compromised” peer review, it is also singled out by Charon Pierson, the-then Secretary of the COPE Trustee Board and Council, as the originary case (Retraction Watch 2016c). In her words, “This case was a major eyeopener for us, as it was the first time we’d ever heard about reviewers setting up dummy email accounts to review their own papers. I think people were jaw-dropped at this; it didn’t even occur to me that it could happen. It represented a turning point for a lot of people about how much journal publishing has changed in the digital era.” According to Pierson, this case ultimately led to changes in COPE’s guidelines for peer reviewers, with the addition of new content on recognizing the possibility of impersonating another individual during the review process (Retraction Watch 2016c). While it is likely that the third case coming so close on the heels of the second influenced COPE’s perception of when the phenomenon emerged, the organization was also unquestionably impacted by Retraction Watch’s coverage of fake reviews during this period.

Retraction Watch started writing about the phenomenon of fake reviews in 2012. The first discussion appeared on Retraction Watch in July 2012 and focused on a Chinese academic who raised the editor’s suspicions when his reviewer suggestions all directed to web domains in China, despite the fact that several listed reviewers were not based there. Treated as an entertaining oddity, the post opens with the line, “Note to authors: If a journal asks you to suggest reviewers for your submitted manuscript, don’t thank them by faking the reviewer’s emails. You might just get caught” (Retraction Watch 2012a). The next case appeared just over a month later and focused on a South Korean plant compound researcher, Hyung-In Moon. This appears to be COPE’s case 12–12 as the details are very similar: the speed at which the reviews were returned concerned the editor and when Moon was contacted, he “admitted to pretending to be some of the peer reviewers that he had ‘recommended’ or to asking colleagues to provide the review” (Retraction Watch 2012c). A further two reports on the same phenomenon were posted the following month: one providing additional evidence of Moon’s transgressions and a fourth piece focusing on Iranian academics. “It’s tempting to start calling this a trend” the latter piece begins (Retraction Watch 2012b).

**Figure 1:**
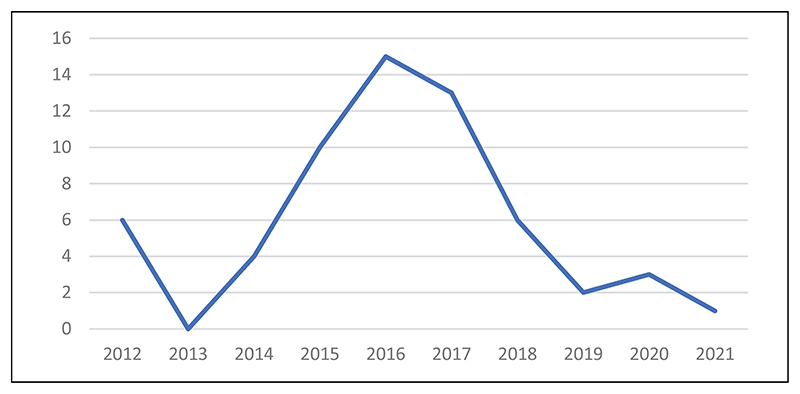
Retraction Watch posts discussing fake reviews.

Although Retraction Watch started tracking fake reviews in 2012, the topic did not feature again in posts until 2014^3^ (see Figure 1), coinciding with the release of their *Nature* news piece titled “Publishing: the peer review scam” (Ferguson, Marcus, and Oransky 2014). The article begins with a discussion of Moon’s case, noting that journal editors are aware of how challenging it is to “persuade busy researchers to review a paper” and highlighting speedy reviews as a warning flag for peer-review “rigging.” “Moon’s was not an isolated case,” the authors warn; “In the past 2 years, journals have been forced to retract more than 110 papers in at least 6 instances of peer-review rigging” (Ferguson, Marcus, and Oransky 2014). Written as an expose of the limitations of automated systems for peer review management, the article highlights “red flags” that signal compromised peer review and quotes the editor in chief of a biomedical journal condemning the practice of allowing authors to recommend reviewers as “bizarre” and “completely nuts.” The piece also mentions that COPE does not have specific guidance on the practice, “but urges journals to vet reviewers adequately” (Ferguson, Marcus, and Oransky 2014).

Shortly after the publication of the *Nature* article, and presumably prompted by it, COPE posted a statement on “inappropriate manipulation of peer review processes,” noting that it had “become aware of systematic, inappropriate attempts to manipulate the peer review processes of several journals across different publishers” (COPE 2014). However, unlike Retraction Watch, COPE treated this primarily as a third-party activity, noting that “some agencies are selling services, ranging from authorship of pre-written manuscripts to providing fabricated contact details for peer reviewers during the submission process and then supplying reviews from these fabricated addresses.” While third party involvement is mentioned in several Retraction Watch posts from this period, it does not feature prominently in their coverage of the topic. Also apparent is a difference in terminology between the two organizations, with COPE more likely to refer to “compromised” peer review or peer review “manipulation” and Retraction Watch more likely to refer to fake reviews.

As Figure 1 indicates, the topic reached its zenith on Retraction Watch in 2016—not coincidentally, the same period when major publishers like Taylor & Francis unilaterally decided to remove the preferred reviewer function from their manuscript submission platforms. Presumably bolstered by the publication of an editorial in the *British Journal of Clinical Pharmacology* on the journal’s recent experience with “organised crime against the academic peer review system” (Cohen et al. 2016), Retraction Watch effectively claimed ownership of the discourse on fake reviews during this period. This is illustrated in the presentation by Retraction Watch’s Alison McCook at a North American COPE seminar in 2016 titled “Can you spot a fake? The trend in fake peer reviews” (COPE 2017). In the presentation, McCook notes that fake reviews are a phenomenon that Retraction Watch has “been tracking for a few years,” framing it as “a new trend in how to subvert the science publishing process.” Describing the issue of fake peer review as “pervading the consciousness of the country” and affecting all major publishers, McCook highlights Retraction Watch’s role in discovering the phenomenon, while acknowledging that COPE became concerned about it “in parallel.” This framing speaks to the broader transformations in science reporting stimulated by the rise of computational and algorithmic journalism, which has rebalanced the relative power of reporters and audiences, restructured business models, and fundamentally changed the content of news itself (Anderson 2012). To quote one of Retraction Watch’s co-founders, “it does not hurt when we talk to funders about the impact we are having on publishing practices and transparency” (Oransky 2020, 141).

In addressing how widespread the problem of fake peer review is, McCook notes that “at least once a week we find a retraction that’s due to some form of compromised peer review… so this is pretty widespread. It’s not just focused on a few bad actors, if you will.” The figures she presents suggest that manipulated peer reviews are indeed a growing problem. In her words, “we’ve counted a total of 319 total retractions due to fake peer reviews since 2012; that’s around a 10th of all papers that have been retracted.” However, toward the end of the presentation, she notes that there are 200 retractions per year, but two to three million papers published: “we only concern ourselves with .03 percent; the vast majority of the literature is fine.” She concludes with the observation that only “0.003 percent” of these papers involve compromised peer review. However, even this figure is somewhat misleading, given that a small number of authors account for the vast majority of retractions involving compromised peer review. Thus, fake peer reviews, by Retraction Watch’s own calculations, constitute an *infinitesimally* small problem in scholarly publishing.

## Staking Out the Fake

According to Retraction Watch’s database, academics are significantly more likely to have had papers retracted for plagiarism or duplication than for fake peer reviews.^4^ However, this is not reflected in Retraction Watch’s coverage of these topics. For example, articles on data fabrication are only slightly more common than articles on fake reviews (a search on the former topic brings up approximately 100 articles). This is partially explained by Retraction Watch’s focus on controversial and newsworthy cases: fake reviews are clearly more attention grabbing than mundane (and arguably more troubling^5^) forms of academic misconduct such as plagiarism and data fabrication. However, it facilitates the misleading impression that fake reviews are a comparatively common form of academic misconduct. More problematically, it serves to reinforce the view that academic misconduct is more common outside the west and the global North, given that most reported cases of fake reviews come from countries such as China,^6^ Iran, and India. Consequently, the fake review discourse tends to implicitly reinforce existing geographies of academic credibility and legitimacy that bisect scholarly publishing^7^ along an implicit North– South divide—themselves connected with longstanding prejudices associating postcolonies with a “counterfeit” rather than “authentic” modernity (Comaroff and Comaroff 2007).

Perhaps paradoxically, the focus on fake peer reviews also serves to shore up the legitimacy of scholarly publishers. Fake reviews clearly pose a genuine threat to publishers because they expose the limitations of their quality controls—especially peer review, with its unique epistemic status in validating manuscripts. Peer review is the key way in which “legitimate” commercial publishers differentiate themselves from the so-called predatory ones, despite their shared profit motive (Bell 2017). Yet fake peer reviews simultaneously pose a threat and an opportunity. While they are a threat to the distinction between legitimate/illegitimate publishers, they (or, more specifically, efforts to combat them through retractions) reaffirm the lines between publishers, providing an opportunity for commercial providers to performatively assert their commitment to ethics and scholarly integrity. Notably, high impact journals have been the most proactive adopters of retraction policies, and the number of journals following suit has dramatically increased in recent years (Fanelli 2020). Thus, retractions for fake reviews or other reasons are a perceived signal of journal quality. Indeed, it is probably not a coincidence that several publishers who carry the taint of the “predatory” label have been particularly proactive in their attempts to crack down on compromised peer review, with retractions leading to publisher-initiated sweeps to check for anomalies (see Retraction Watch 2015; Retraction Watch 2016a).

**Figure 2:**
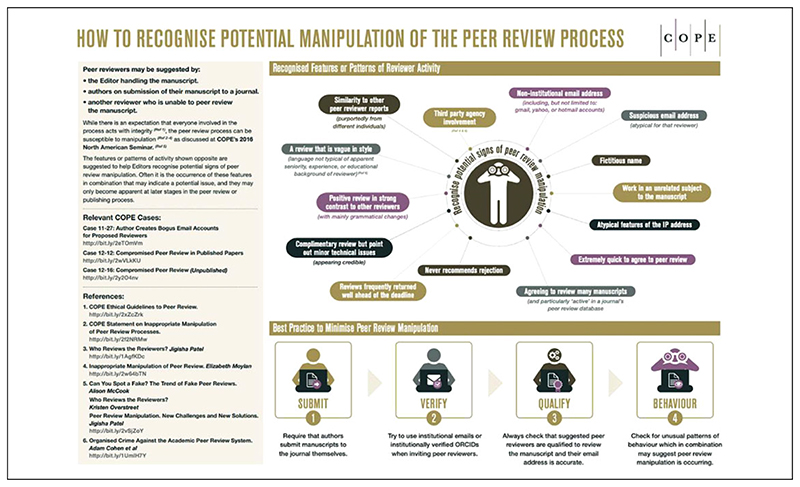
COPE infographic on spotting peer review manipulation (reproduced under CC-BY license).

Articles posted on Retraction Watch about retractions on the basis of peer review manipulation are replete with statements from publishers framing themselves as “custodians” and “guardians” of research integrity. Notably, it is usually a spokesperson from the publisher, rather than the journal editor in question, making these statements. Publishers frequently highlight the taskforces they have created, investigations they have launched, and, on occasion, the journal editors they have sanctioned. For example, a post regarding a series of retractions in *Technology in Cancer Research & Treatment* includes a statement from a Sage employee explaining that she has replaced the editor-in-chief and is “working closely with a team of Associate Editors to manage peer review” (Retraction Watch 2017c). Indeed, it is clear that editors are perceived ambivalently in the discourse on fake reviews.^8^ Recall that COPE specifically highlights the responsibility of editors for failures in their peer review processes, placing considerable onus on editors to identify and deal with fake reviews, as their “How to recognise potential manipulation of the peer review process” infographic (Figure 2) and their “Peer review manipulation suspected during the peer review process” algorithm (COPE 2019) attest. Publishers themselves are almost entirely absent from COPE discussions of peer review manipulation.^9^ This contrasts with their hyper-visibility on Retraction Watch as defenders of scholarly integrity. In effect, the fake review discourse allows publishers to simultaneously disavow responsibility for the problem, while demonstrating the steps they are taking to police it. Thus, in a landscape where journals are vying for credibility in the face of widespread concerns about prestige and quality, signifiers of credibility translate into improved revenues for publishers. Swift action in the face of fake reviews thereby serves a useful marketing function.

This is not to suggest that there are no detrimental consequences for publishers posting article retractions for fake reviews; instead, publishers seek to manage retractions in a way that enhances their reputation rather than undermining it. A case in point is Retraction Watch’s coverage of Web of Science’s decision to stop indexing *Tumor Biology,* a journal that retracted 107 papers for faked peer reviews. Titled “When a journal retracts 107 papers for fake reviews, it pays a price,” the article emphasizes that without indexation in Web of Science, the journal will lack an impact factor, “which can be the kiss of death for many journals” (Retraction Watch 2017d). While Retraction Watch focuses on how fake reviews threaten publishers, the phenomenon poses a striking opportunity for commercial indexes like Web of Science to performatively assert *their own* commitment to quality and integrity in an environment where it, too, has been challenged by the appearance of rival indexes and critiques of their reliability as arbiters of journal quality (see Archambault and Larivière 2009; Archambault et al. 2009). To quote from a Clarivate Analytics spokesperson, “After a thorough review, Clarivate Analytics has deselected *Tumor Biology* from continued coverage in Web of Science. This journal no longer meets the high standards required for inclusion due to the severity of peer review malpractice” (Retraction Watch 2017d).

Notably, other commercial players in the scholarly publishing ecosystem have also seen the potential of the fake review discourse in promoting their own services. For example, in 2017, Retraction Watch featured an interview with the co-founder and CEO of Publons, a database of vetted peer reviewers that publishers can pay to access, about how the database can help curtail the problem of fake peer reviews^10^ (Retraction Watch 2017a). Likewise, that same year it featured an interview with the Head of Product for the ScholarOne manuscript submission platform about its new tools for identifying fake reviews (Retraction Watch 2017b). In these accounts, fake peer reviews are primarily a technical problem that can be resolved through more diligent training of editors, databases of vetted reviewers, and better technology to pick up and rectify problems.

## Fake versus Genuine Peer Review

Although Retraction Watch and COPE treat peer review as a primarily technical problem, they inadvertently raise a number of intriguing questions about the boundary between “authentic” or “genuine” and “fake” peer review. The clearest illustration of the issues raised by peer review can be found in a Retraction Watch post on computer-generated fake reviews (Retraction Watch 2016b). The post takes the form of an interview with Eric Medvet regarding a tool created by a team of researchers to test the feasibility of computer-generated peer reviews (see Bartoli et al. 2016). The researchers found that even experienced academics could not always tell the difference between fake and genuine peer reviews, suggesting that the lines between them are far from clear cut. Indeed, scrutiny of COPE and Retraction Watch’s websites reveals that constant boundary management is required to maintain the lines between the two. This is evident in responses to the COPE Forum case (12–12) that seems to have catalyzed the fake peer review discourse in the first place. To quote “Dodgy Geezer’s” observations around the practice of using close associates as reviewers, “But this is pretty normal practice in Climate Science. Indeed, the tight circle of ‘reviewer pals’ was outed by the Wegman report” (COPE 2012). While we do not intend to relitigate the legitimacy of the peer review practices exposed by “Climategate,” the debates that followed demonstrate a lack of agreement among scientists on whether these practices were outside the bounds of “normal” scientific behavior^11^ (see Grundmann 2012).

Clearly, the independence of peer review is not straightforward in many fields. For example, in particle physics, where authorship lists regularly run into the hundreds, independent peer review is an impossibility: those with the expertise to review papers are already likely to be authors on them (see Galison 2003). Moreover, in fields where rigor is conceptualized more in terms of authors’ grasp of the theories and methods they have employed than the reproducibility of their data, far from being “bizarre” and “completely nuts,” the practice of recommending reviewers is based on the expectation that those best placed to review papers are sympathetic to, and therefore knowledgeable about, the theories and methods being employed. Problems arise in such contexts primarily when reviewers are allocated *without* due consideration of their disciplinary background and methodological expertise. Indeed, there is evidence that attempting to limit bias by privileging impartiality over expertise reduces the quality of the peer review process (Li 2017). Conversely, a scientist is hardly likely to get a fair hearing from a peer fundamentally opposed to their ideas and arguments or whose own research findings are challenged by them—a possibility acknowledged in the practice of allowing “non-preferred” as well as “preferred” reviewers.

Beyond the question of the bias of peer reviewers, several of the red flags listed by COPE and Retraction Watch will be familiar to any journal editor exposed to legitimate peer reviews (and, indeed, any published academic), such as “a review that is vague in style” and “complimentary review but point out minor technical issues” (see Figure 2). The fact is that vague, unhelpful, and poorly written peer reviews are reasonably common, something COPE readily acknowledges elsewhere on its website, such as in its case discussion on “Editing peer review comments” (COPE 2020). Anyone who has ever received a peer review will likely have had cause to question whether a particular reviewer has actually read their paper. Indeed, in the interview with Retraction Watch quoted earlier, Eric Medvet speculates that those interested in generating fake reviews might include, “scholars who want to take part in many program committees or editorial boards, just to earn the corresponding credits without actually spending time in reviewing papers” (Retraction Watch 2016b). Where does a fake review written by a real reviewer fit into the fake versus genuine review dichotomy? And, in these circumstances, how is a fake review different from a poor one? In our view, the terminology of the fake has served to obscure the vexed philosophical and existential questions that this phenomenon raises about the nature of peer review. After all, the differences between a non-peer review and a peer non-review^12^ are negligible in terms of their stated function to epistemologically validate manuscripts.

The broader issues raised by peer review manifest most palpably in the figure of “Reviewer 3” (or, in some accounts, “Reviewer 2”), immortalized in endless memes, cartoons, and Facebook pages (e.g., “Reviewer 2 Must Be Stopped”), along with the popular Tumblr account “Shit My Reviewers Say.” This reviewer, to quote one succinct definition, “is the embodiment of all that is wrong with the peer review system. No, wait, all that is wrong with the *peer* in peer review” (Brown 2015, emphasis in original). According to most accounts, they are the petty, narcissistic, incompetent reviewer who holds hapless academics hostage to their mercurial, unreasonable whims—an “asshole,” in so many words (Brown 2015). The folklore surrounding Reviewer 2/3 is so pervasive that it is beginning to crop up in serious academic commentaries (e.g., Ingber 2020), and satiric scholarly studies such as “Dear Reviewer 2: Go F’ Yourself” (Peterson 2020). While Reviewer 2/3 is primarily treated as an academic meme, we are fascinated by the deeper anxieties this figure reveals about peer review.

In Retraction Watch’s initial post on the Korean plant compound researcher Hyung-In Moon, which is widely seen to have ushered in the discourse on fake reviews, such reviews were presented as a conceivable *response* to the problem of the third reviewer. “Scientists frustrated by the so-called ‘third reviewer’—the one always asking for additional experiments before recommending acceptance—might be forgiven for having fantasies of being able to review their own papers,” the post begins (Retraction Watch 2012c). This implicitly highlights the role of Reviewer 3 in *authenticating* the peer review process. After all, a key warning flag for fake peer reviews highlighted by both COPE and Retraction Watch is uniformly positive reports. To quote Retraction Watch’s Alison McCook: “When all three reviewers like the paper, that’s when you’ve got to kind of, you know, like, be a little suspicious” (COPE 2017).

Yet, in the folklore on Reviewer 3, the third reviewer is often itself treated as suspicious—a view encapsulated in the suggestion that Reviewer 3 is frequently a ghost-writer. In such accounts, Reviewer 3 is deemed to be either a journal editor using a forged report to justify their negative editorial decision or an angry postdoctoral researcher forced to write uncredited reviews for their dissertation supervisor (see Vines 2012). Although the reviewer’s identity has been actively disguised and misrepresented in both compromised reviews and ghost-written ones, their contrasting perception is instructive. The value of ghost-written reviews is not generally in question, despite raising the specter of misrepresentation (when an editor writes peer reviews and passes them off as third-party assessments) and exploitation (when postdocs write uncredited reviews). This is primarily because they do not subvert the function of peer review as a form of “institutionalized epistemic vigilance” (Gross and Harmon 2016, 127).

In light of the distinctive role of editors in organizing and curating the peer review process (see Kaltenbrunner et al. 2021), bias in editorial decisions only becomes described in terms of misconduct when it is *in favor* of authors for the editor’s material gain (see Petersen 2019). This suggests the value placed upon peer review as a form of gatekeeping rather than quality control. In effect, we are witnessing how peer review sustains what Eve (2013) has referred to as a “zombified” system of scholarly publishing, whereby the “no-brainer” logic of selecting the most prestigious publishing outlet makes that outlet simultaneously less accessible and more desirable in a perpetual feedback loop. This feedback loop, as Eve notes, has served to drive up the subscription prices of the most desirable outlets, while creating a situation where the volume of publications submitted greatly outstrips the capacity of available peer reviewers. In fact, increasing rates of desk rejections are directly related to the undersupply of reviewers in relation to the oversupply of papers (see Garand and Harman 2021). In this environment, the gatekeeping function of peer review has been amplified and conflated with its role as a form of quality control.^13^

## Discussion

Despite the rarity of compromised peer review, the discourse on peer-review fakery has indelibly altered scholarly publishing—especially how manuscripts are processed behind the scenes. The effects of this crisis discourse reveal the risks of a loss in trust in the review process, highlighting the significance of peer review as a form of boundary work that distinguishes science from nonscience and also as a process that spans the boundaries of numerous social worlds (Chubin and Hackett 1990). Clearly, a variety of players in the scholarly publishing ecology have strategic, commercial, and cultural interests in peer review and the critical symbolic and ideological function it performs: scholars, the academy, and research funders, along with publishers, indexes, and other infrastructure providers, and a growing array of self-declared “watchdogs” of scholarly publishing ethics and integrity. Indeed, without Retraction Watch’s interventions, it is unclear to us whether a fake review discourse would have emerged at all, which speaks to the ways in which the digital era has spawned radical transformations in how scientific knowledge is “generated, communicated, and evaluated” (Gross and Harmon 2016, 11).

While the epistemological function of peer review remains central in validating publications, it has taken on additional functions in validating publishers in a crowded and heavily commercialized marketplace. In effect, peer review serves to authenticate a system with conflicting demands over value (Jacob 2020) and divergent conceptions of what that entails. For commercial publishers, the value of peer review is its role as a market mechanism in differentiating journals. This is evident in the decision numerous publishers made to unilaterally remove the preferred reviewer function from their submission platform, even though it arguably improves the quality of peer reviews when employed appropriately.^14^ In a political-economic environment primed by alarmism around fake reviews, being seen to do something was more important than actually fixing the problem, which, after all, would require a consideration of the dynamics of the relationship between the academy and publishing itself, from the sources of misconduct to the overproduction of publications to the reasons for the contemporary peer review*er* crisis.^15^

As anthropological work on the fake has shown, discourses of fakery require that objects or practices are repositioned within a simplified and flattened moral landscape (Yurchak 2018; Beek et al. 2019). Beek et al. (2019, 427) argue that “practices become fraudulent and objects fake by actors producing them as such” through suspicion and distrust. Thus, any normative account of fakery and fraud “not only describes but also *partakes in the making of fraud”* (429, emphasis added). This is because discerning fakes and frauds is more than a practical or technical problem. It raises complex and often intractable philosophical and conceptual questions that become readily evident as soon as one looks beneath the surface of the distinction between genuine and fake reviews, where we find that these categories are far more contingently constructed than they first appear (cf. Jacob 2020). Although we are not intending to downplay the seriousness of attempts to intentionally subvert and manipulate the peer review process, as we have illustrated, constant work is required to maintain clear lines of separation, especially given the intrinsic ambiguities and suspicions surrounding the nature of the peer review process.

The notion of the fake review is sustained by a larger conceptual dichotomy between facts and values that has been heavily critiqued by scholars of science (see especially Latour 1993). In this framing, “‘facts’ are epistemic objects discovered by scientists that are amenable to a process of peer evaluation; ‘values’ are subjective expressions of personal preference” (Frodeman and Biggle 2012, 16). Indeed, a key feature of Retraction Watch is that it never probes beneath the surface of the retractions it covers to consider science as a social institution and the tensions between its incentives and norms (Gross and Harmon 2016). As Jacob (2020, 256) observes, “Whilst concerned with hunting misconduct, watchdogs pay less attention to the systemic features of the mainstream science on which the counterfeit models itself.” Yet the purity of peer review is always in question—even more so in the 21st century, given its “de-disciplining” via experiments in new forms of review. In the words of Frodeman and Biggle (2012, 4), “Peer review is increasingly being called on to do what Latour described as the work of translation—the contrary of purification, as it creates science–society mixtures or hybrids.”

Amid such destabilization and growing questioning among academics about the value and purpose of peer review, the fake review discourse has been instrumental in reasserting the boundaries between science and society and reaffirming the ideological and symbolic significance of the so-called genuine prepublication peer review. As Jacob (2020) observes,

Counterfeiting solidifies the “template” of elite science and keeps it intact; in other words, by reinforcing the value and prestige of the model,… counterfeiters possibly contribute to sustain the structures of mainstream science by keeping them intact and off the radar whilst our scrutiny targets the counterfeiters. Further, through distinguishing themselves from the counterfeiter, the counterfeited… accumulates further symbolic capital. (256)

Indeed, the stakes in maintaining these distinctions are arguably higher than ever, given the ways in which pre-publication review has become tied to the production and processing of papers as currency for a commodified research enterprise. In this environment, the discourse of peer-review fakery obviously presents reputational and financial risks for commercial publishers and other infrastructure providers—but new opportunities for market segmentation as well. This speaks to the ongoing salience of Chubin and Hackett’s observation more than thirty years ago that “while the peer review *system* may absorb severe damage, the peer review *concept* emerges with renewed support from all parties” (1990, 5, emphasis in original). This situation seems unlikely to change in the absence of a radical material and ideological reconceptualization of scholarly publishing and greater recognition of precisely how the meanings of peer review have been transformed in the twenty-first century.

## Footnotes

1 Notes Retraction Watch’s funding has come primarily from the Arnold Foundation, which is committed to replicable science and exposing poor scientific practice.

2 This is the case typically referenced in COPE’s materials as its initial exposure to compromised peer review.

3 Importantly, Retraction Watch’s coverage of fake reviews on their website is not a straightforward reflection of their database. Their database indicates fifteen retractions for fake reviews in 2013, although there was no coverage of this topic in their blog posts that year. Moreover, while Retraction Watch’s coverage of fake reviews fell after 2016, retractions due to fake reviews remain high, with 204 retractions in 2016, 155 in 2017, 87 in 2018, and 119 in 2019.

4 As of March 29, 2021, the database included 26,806 retractions, 940 of which are categorized under “fake reviews.” To contextualize this figure, there were 3,897 retractions categorized under “plagiarism” or euphemisms for such and 4,316 retractions categorized under “duplication” or euphemisms for such.

5 It is worth emphasizing that the findings of these retracted papers are not necessarily in dispute, just the peer review process itself. In many instances, co-authors approached by Retraction Watch have indicated that they were unaware of any peer review rigging on the part of the submitting author and highlight the devastating impacts on their careers as a result of their presumed guilt by association.

6 According to Retraction Watch (2017c), China is the leading national producer of fake reviews. However, according to Retraction Watch’s database, China has had more retractions than any other country for *all* forms of academic misconduct, which is often attributed to the use of institutional incentives such as cash bonuses for publications in high-ranking journals (see Quan, Chen, and Shu, 2017).

7 In this respect, it functions similarly to the discourse on “predatory” publishers. Elsewhere, we have highlighted the way in which the label reifies a false dichotomy between “legitimate” (established Northern) publishers and “illegitimate” (emerging Southern) ones (Bell 2017; Mills et al. 2021).

8 The concern about fake reviews appears to be expediting the professionalization of the journal editor role, with the traditional university-based academic editor increasingly side-lined in favor of a paid executive editor employed by the publisher and often lacking any specific research expertise in the subject matter of the journal, but well versed in “best practice” in scholarly publishing. This constitutes a highly significant—albeit largely unremarked—transformation in the role of the journal editor that speaks to the ways in which editors are now expected to curate journals as “brands with a value indicated by their impact factor and the level of income they generate for commercial publishers, and intellectual homes of communities of scholars” (Bell et al. 2021, 379).

9 In some respects, this is not surprising, given that COPE was set up by a group of journal editors and most of its cases deal with the issues of direct concern to editors themselves. However, as its stated aim is to support editors, publishers, and “those involved in publication ethics,” the absence of consideration of the responsibilities of publishers is striking.

10 Publons relies on academics’ interest in receiving credit for their otherwiseinvisible peer review labor, with the resultant database now made commercially available to publishers and integrated with the ScholarOne platform—made possible by the fact that Clarivate Analytics owns both companies. Clearly, various commercial interests have been quick to capitalize on the fake review discourse.

11 In this respect, the discourse on “peer review rings” suffers the same problems as the discourse on fake reviews because it implies a clear line of separation between regular and rigged reviews that cannot be so readily sustained in practice.

12 We are grateful to one of the reviewers for this observation.

13 Rejection rates are one of the criteria Web of Science use in assessing the suitability of journals for indexation in its database.

14 In contexts where the preferred reviewer option has been reinstated, reviewers are typically required to have an institutional email address, causing problems when (legitimate) prospective reviewers are retired, between academic positions, or otherwise prefer to use non-institutional email addresses—a common practice outside the global North.

15 Notably, the picture presented of peer reviewers in COPE’s and Retraction Watch’s accounts of how to spot fake peer reviews is of a surly, chronically overworked academic, with reviewers returning positive and speedy peer reviews thereby treated as automatically suspect.
